# Distributed Algorithm for Voronoi Partition of Wireless Sensor Networks with a Limited Sensing Range

**DOI:** 10.3390/s18020446

**Published:** 2018-02-03

**Authors:** Chenlong He, Zuren Feng, Zhigang Ren

**Affiliations:** 1 State Key Laboratory for Manufacturing System Engineering, Systems Engineering Institute, Xi’an Jiaotong University, Xi’an 710049, China; fzr9910@mail.xjtu.edu.cn; 2 Autocontrol Research Institute, Xi’an Jiaotong University, Xi’an 710049, China; renzg@mail.xjtu.edu.cn

**Keywords:** distributed algorithm, limited Voronoi partition, local information, Wireless Sensor Networks (WSNs), sensing capability

## Abstract

For Wireless Sensor Networks (WSNs), the Voronoi partition of a region is a challenging problem owing to the limited sensing ability of each sensor and the distributed organization of the network. In this paper, an algorithm is proposed for each sensor having a limited sensing range to compute its limited Voronoi cell autonomously, so that the limited Voronoi partition of the entire WSN is generated in a distributed manner. Inspired by Graham’s Scan (GS) algorithm used to compute the convex hull of a point set, the limited Voronoi cell of each sensor is obtained by sequentially scanning two consecutive bisectors between the sensor and its neighbors. The proposed algorithm called the Boundary Scan (BS) algorithm has a lower computational complexity than the existing Range-Constrained Voronoi Cell (RCVC) algorithm and reaches the lower bound of the computational complexity of the algorithms used to solve the problem of this kind. Moreover, it also improves the time efficiency of a key step in the Adjust-Sensing-Radius (ASR) algorithm used to compute the exact Voronoi cell. Extensive numerical simulations are performed to demonstrate the correctness and effectiveness of the BS algorithm. The distributed realization of the BS combined with a localization algorithm in WSNs is used to justify the WSN nature of the proposed algorithm.

## 1. Introduction

In the last few decades, Wireless Sensor Networks (WSNs) have been widely used in practical applications, where a large number of simple individuals is organized into a network to perform some sophisticated tasks effectively, such as the cooperative exploration of an unknown terrain [1], the optimal coverage of a region [2] and the distributed deployment of a multi-robot system [3]. In these applications, individuals having the sensing and moving ability are generalized into sensors. The environment monitored by sensors should be divided into a collection of disjoint regions, each of which is dominated by only one sensor. A feasible technique to generate region divisions is known as the Voronoi partition or Voronoi tessellation.

The concept of the Voronoi partition comes from computational geometry. It is commonly used to separate a plane into multiple regions by a collection of points called generators. For each generator, its dominant region is called the Voronoi cell consisting of all points closer to itself than to any other [4]. Traditional algorithms used to compute a Voronoi partition are implemented in a centralized manner [5], where the position information of all the points is used to generate the entire Voronoi diagram. However, the distributed and resource-constrained nature of WSNs invalidates these centralized algorithms. Therefore, it is necessary to devise a correct and efficient algorithm for each sensor to autonomously compute its own Voronoi cell based on the relative positions of the nodes within its limited sensing or communication range so that the complete Voronoi partition of the WSN can be generated in a distributed fashion.

Besides WSNs, a similar problem of the Voronoi partition also lies in other distributed systems such as multi-agent systems and robotic networks, where the information exchange among individuals is indispensable for completing the task cooperatively. In [6], a distributed algorithm was proposed for multi-robot systems to compute the Voronoi partition by assuming that each robot as a mobile sensor can obtain the position information of all the others. Through enlarging a virtual sensing radius, the initially approximate Voronoi cell is gradually refined to the exact one. A distributed algorithm used to obtain the Voronoi diagram for a WSN was proposed in [7], where the information exchange was realized by means of communication among sensors. The exact Voronoi diagram is computed by trivial geometric operations of half-plane intersections. Similarly, in [8], each sensor acquired sufficient information to construct its Voronoi cell by means of the multi-hop communication from its neighbors. The core of the above two algorithms is the design of an appropriate communication protocol among sensors.

For the WSN consisting of sensors having a passive sensing device with a limited sensing range or an active communication device with a power constraint, it is impossible to compute the exact Voronoi cell for each sensor with insufficient information. Instead, the exact Voronoi cell is approximated by the Voronoi cell within a constrained circle, which is called the range-limited Voronoi cell [9]. Theoretically, in order to generate a correct Voronoi tessellation of a WSN in a distributed way, each sensor should construct its own limited Voronoi cell within a constrained range based on the position information of other sensors lying in a circle with a radius at least double the constrained range.

The most commonly-applied algorithm for computing the Voronoi partition is the classical Adjust-Sensing-Radius (ASR) algorithm proposed in [10]. Without considering the constraint on the sensing range, the exact Voronoi cell of the sensor can be gradually refined by computing the Voronoi cell within an adjustable sensing circle and enlarging the virtual sensing range iteratively. Furthermore, this algorithm can be trivially generalized to compute the range-limited Voronoi cell [9,11] and has been regarded as a standard algorithm to solve the problem of the Voronoi partition in a distributed manner over the last few decades.

However, in the ASR algorithm, there is a key step involved in computing the limited Voronoi cell in the virtual sensing range, which is implemented by the definition of the limited Voronoi cell, i.e., first computing the full Voronoi partition of the node set consisting of the generator and its neighbors within the virtual sensing range by a centralized algorithm of the Exact Voronoi Tessellation (EVT), then extracting the exact Voronoi cell of the generator, finally intersecting the Voronoi cell with the constrained circle, whose radius is equal to the current adjustable sensing range. Although the computation can be accomplished by existing Voronoi tessellation techniques in a centralized manner and has an optimal computational complexity, it is not the most efficient method to generate the limited Voronoi cell, since the result produced by the centralized algorithm includes not only the necessary Voronoi cell of the generator, but also the unnecessary ones of its neighbors. When the ASR algorithm is generalized to compute the limited Voronoi cell, the lack of efficiency to compute the limited Voronoi cell in the key step still limits its applicability.

An algorithm called the Range-Constrained Voronoi Cell (RCVC) algorithm was proposed in [12] to efficiently compute only the range-limited Voronoi cell of a robot having a limited sensing range through comparing a collection of chords, which are the bisectors between the robot and its neighbors in the communication range twice its sensing range. Although this algorithm generates just the limited Voronoi cell belonging to the generator, it has a relatively high computational complexity in its “removing redundant chords” subroutine, which actually has a quadratic rather than a linear computational complexity stated in that paper.

In this paper, we propose an efficient algorithm for each sensor with the same and limited sensing range to compute its limited Voronoi cell autonomously so that the limited Voronoi partition of the entire WSN is generated in a distributed manner. In the proposed algorithm, each sensor should be able to acquire the position information from its neighbors in the sensing range to compute the candidate boundary set of the limited Voronoi cell, i.e., bisectors between itself and its neighbors within the half sensing range. Inspired by the classic Graham’s Scan (GS) algorithm [13] used to construct the convex hull of a point set, all the boundaries of the limited Voronoi cell are determined by sequentially scanning the collection of bisectors sorted by directional angles and comparing the relative position of two consecutive bisectors in the proposed algorithm. Thus, this algorithm is called the Boundary Scan (BS) algorithm. The BS algorithm has a lower computational complexity than the RCVC algorithm and reaches the lower bound of the computational complexity to solve problems of this kind. Furthermore, the BS algorithm is not only an improvement of the key step in the ASR algorithm, but also more time-efficient than the whole ASR algorithm for low-density WSNs. Thus, the relevant research such as the coverage control for sensor networks [14,15], the coordination of multi-robot systems [16] and node-to-actor allocation algorithm for wireless sensor and actor networks [17], where the construction of the Voronoi partition is the crucial technique, will benefit by the computational efficiency of the BS algorithm. Finally, extensive numerical simulations are carried out to verify the correctness and effectiveness of the proposed algorithm. The distributed realization of the BS combined with the Distance Vector Hop (DV-Hop) algorithm [18], which is a widely-used localization algorithm in WSNs, is implemented to justify the WSN nature of the proposed algorithm.

This paper is outlined as follows. In Section 2, the definition of the Voronoi diagram, as well as some relevant concepts are introduced. Then, the problem of computing a limited Voronoi cell is formulated. Section 3.1 gives the basic representations of the BS algorithm. The descriptions and operations of three steps in the BS algorithm are elaborated in Section 3.2. In Section 3.3, the similarity of the BS and GS algorithm is stated to indicate the feasibility of the BS algorithm. The computational complexity is analyzed in Section 3.4. In Section 4, numerical simulations are performed to justify the correctness, effectiveness and applicability of the BS algorithm. The conclusion and future work are given in Section 5.

## 2. Problem Formulation

For a WSN consisting of *n* homogeneous sensors with a fixed and limited sensing range, the configuration of the network is denoted by a collection of position coordinates of sensors in a plane $P=\{p_{i}\in R^{2},i\in I_{n}\}$, where $I_{n}=\left\{1,\ldots ,n\right\}$ is an index set. In what follows, we shall use the position $p_{i}$ to indicate the *i*-th sensor. The half plane closer to $p_{i}$ than to $p_{j}$ is denoted as:

$$\begin{array}{c} H\left(p_{i},p_{j}\right)=\{q\in R^{2}|∥p_{i}-q∥\leq ∥p_{j}-q∥\}. \end{array}$$

The Voronoi cell of $p_{i}$ is defined as the intersection of all the half planes closer to $p_{i}$ than to any other sensors as:

(1) $$\begin{array}{c} V_{i}\left(P\right)=\{q\in R^{2}|∥p_{i}-q∥\leq ∥p_{j}-q∥,j\in I_{n}\setminus \left\{i\right\}\}. \end{array}$$

The sensor $p_{i}$ is called the generator of its Voronoi cell $V_{i}\left(P\right)$. The Voronoi diagram or Voronoi partition generated by a wireless sensor network consists of all the Voronoi cells of sensors as $V\left(P\right)=\{V_{i}\left(P\right),i\in I_{n}\}$.

For sensor $p_{i}$ and $p_{j}$, if their corresponding Voronoi cells share a common boundary, i.e., $V_{i}\left(P\right)⋂V_{j}\left(P\right)\neq \emptyset$, then two sensors become Delaunay neighbors, all of which form the edge set of the Delaunay graph (denoted as $G_{D}$). Since the Delaunay graph is dual to the Voronoi diagram, it is easy to derive one graph from its dual counterpart [19]. We shall alternatively use these two representations in the following paper.

There are generally two ways for nodes of the WSN to acquire the position information from their neighbors. For one thing, if a node is equipped with a passive sensing device, such as infrared, ultrasonic or laser, it will obtain the position information of its neighbors by means of direct sensing. For another, if a node has an active communication function, the position information will be transferred among nodes by one-hop communication. In this paper, it is assumed that each node collects necessary information by means of sensing. The limited sensing range of a node is represented as a circle. For sensor $p_{i}$, its sensing range is denoted as a closed circle $\bar{B}\left(p_{i},r\right)$ with the center $p_{i}$ and radius *r*. If sensor $p_{j}$ lies in the sensing range of sensor $p_{i}$, i.e., $p_{j}\in \bar{B}\left(p_{i},r\right)$, then $p_{i}$ and $p_{j}$ are neighbors of each other, and there is an undirected edge between them, i.e., $\left(i,j\right)\in E_{G_{disk}\left(r\right)}\Leftrightarrow \left(j,i\right)\in E_{G_{disk}\left(r\right)}$, where the graph is called the *r*-disk graph (denoted as $G_{disk}\left(r\right)$). The neighbor set of sensor $p_{i}$ in the *r*-disk graph is denoted as:

$$\begin{array}{c} P_{i}^{r}=\{p_{j}\subseteq P|∥p_{i}-p_{j}∥<r,j\in I_{n}\setminus \left\{i\right\}\}. \end{array}$$

**Figure 1:**
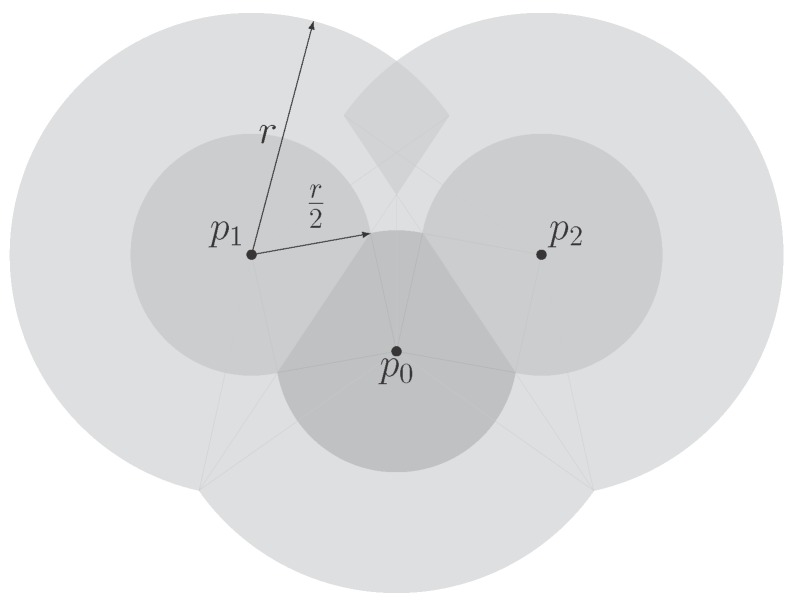
The comparison of the valid and invalid approximate Voronoi partition of three sensors with a limited sensing range *r*.

Within the context of insufficient information, the exact Voronoi cell, which is not generally obtained, should be intuitively approximated by the part of the exact Voronoi cell that lies within the sensing range of the node. However, such approximate Voronoi cells of all the sensors do not constitute a valid partition of a plane due to the overlap of the interior of these regions. The reason why the invalid partition occurs is that some boundaries separating two Voronoi cells lie outside the sensing range and cannot be perceived by sensors. In Figure 1, two limited Voronoi cells within the respective sensing ranges of sensor $p_{1}$ and $p_{2}$ are overlapped (shown as the light gray regions in the figure) since two sensors are not neighbors in the *r*-disk graph. In contrast, the approximate Voronoi partition within the half sensing range (shown as the dark gray regions in the figure) is valid since the bisector between two non-neighboring sensors is outside the constrained circle, whose radius is equal to the half sensing range.

The aforementioned *r*-disk and Delaunay graph are known as proximity graphs in computational geometry. A proximity graph is composed of a vertex set, which is a collection of distinct points, and an edge set, which is a set representing the adjacent relations determined by the relative locations of point pairs. Theoretically, Proposition 2.9 in [9] gives the derived relationship among different proximity graphs, which is generalized into the spatially-distributed property. Here, we only focus on how to derive the best approximate Voronoi diagram with the constraint of a limited sensing range. Therefore, the *r*-limited Delaunay graph (denoted as $G_{LD}\left(r\right)$), which is spatially distributed over the *r*-disk graph, will meet our requirement. This means that the *r*-disk graph encodes sufficient information to compute the *r*-limited Delaunay graph, which is dual to the r/2-limited Voronoi diagram consisting of multiple r/2-limited Voronoi cells of all the sensors. Take sensor $p_{i}$ for example; its r/2-limited Voronoi cell is denoted as:

(2) $$\begin{array}{c} V_{i}^{r/2}\left(P_{i}^{r}\right)=V_{i}\left(P_{i}^{r}\right)\cap \bar{B}\left(p_{i},r/2\right), \end{array}$$

i.e., the intersection of the exact Voronoi cell and the constrained circle whose radius is equal to the half sensing range of a sensor. Therefore, the plane to be partitioned by a WSN with a limited sensing range is not the entire plane, but the union of all the half sensing circles as $\cup_{i\in I_{n}}\bar{B}\left(p_{i},r/2\right)$.

**Figure 2:**
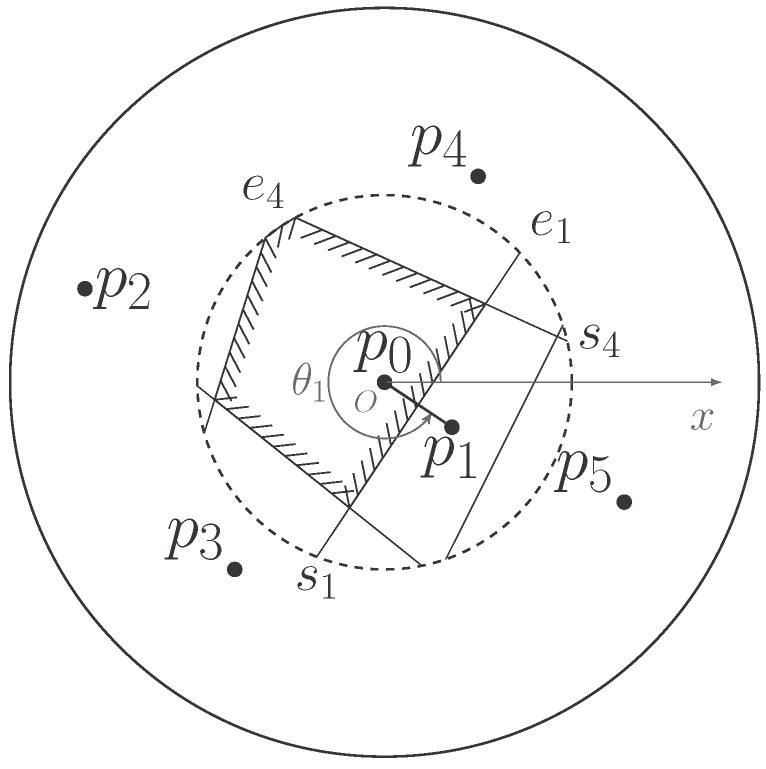
The r/2-limited Voronoi cell of sensor $p_{0}$ along with other notations.

Figure 2 illustrates a six-node configuration as an example of a WSN and highlights the r/2-limited Voronoi cell of sensor $p_{0}$. This region is enclosed by shaded line segments, which are part of the bisectors between $p_{0}$ and other sensors in its sensing range depicted as the big circle with a solid line as its circumference and shaded arcs belonging to the constrained circle depicted as the small circle with a dashed line as its circumference. Thin line segments are the bisectors inside the constrained range. They are also the boundaries of the half planes between the sensor and its neighbors. The shaded side of the boundaries is the half planes in which sensor $p_{0}$ is located. Two extreme points of the bisector between $p_{0}$ and $p_{1}$ are exclusively marked as $s_{1}$ and $e_{1}$ for later use. They are also the starting and ending points of the arc on the constrained circle. According to the relative position of these bisectors, it is evident that the bisector between $p_{0}$ and $p_{5}$ is not the boundary of $p_{0}$’s Voronoi cell, so $p_{5}$ does not belong to the *r*-limited Delaunay neighbor set of $p_{0}$.

## 3. Description and Analysis of the Boundary Scan Algorithm

### 3.1. Representation of Basic Elements in the Boundary Scan Algorithm

Due to the distributed property of WSNs, the r/2-limited Voronoi partition of a plane should be decomposed autonomously computing the r/2-limited Voronoi cell of each sensor by means of the BS algorithm. In the implementation of the BS algorithm, a local Cartesian coordinates system is set up at the position of the generator, and its neighbors are represented in the local coordinates. Intermediate results of the BS algorithm including the relative information between the generator and its neighbors, such as their distances and directional angles, and final results recorded as a collection of vertices of the r/2-limited Voronoi cell do not depend on the global coordinates system.

For ease of comparing the relative position of two bisectors, each bisector is represented as a directed line segment with two ordered extreme points. Taking sensor $p_{i}$ for example, all bisectors between it and its neighbors make up the bisector set $B_{i}=\left\{{\vec{b}}_{j},j\in I_{m}\right\}$, where $m=|P_{i}^{r}|$. The element of the set is denoted as ${\vec{b}}_{j}=s_{j}e_{j}$, which indicates that the directed line segment has a starting point $s_{j}$ and ending point $e_{j}$ such that along the direction from $s_{j}$ to $e_{j}$, the generator $p_{i}$ lies on the left of the line segment. Meanwhile, the polar angle $\theta_{j}$ of the sensor $p_{j}$ in the polar coordinates of sensor $p_{i}$ is regarded as the directional angle of the bisector ${\vec{b}}_{j}$ for sorting the bisector set.

Seeing in Figure 2 again, the bisector ${\vec{b}}_{1}$ between sensor $p_{0}$ and $p_{1}$ is represented as the directed line segment from the starting point $s_{1}$ to the ending point $e_{1}$. The left side of ${\vec{b}}_{1}$ is the half plane dominated by $p_{0}$. The directional angle $\theta_{1}$ of ${\vec{b}}_{1}$ is the polar angle of point $p_{1}$ in the polar coordinates with $p_{0}$ as the pole and the horizontal axis as the polar axis. With the above representations, the relative position between two bisectors can be readily determined by the cross product of the directed line segments formed by the extreme points of two bisectors. Taking bisector ${\vec{b}}_{1}$ and ${\vec{b}}_{4}$ for example, for the starting point of ${\vec{b}}_{4}$, the result of the cross product between two directed line segments $s_{4}s_{1}$ and $s_{4}e_{1}$ is less than zero, which indicates that $s_{4}$ is on the right of ${\vec{b}}_{1}$. Likewise, $e_{4}$ is on the left of ${\vec{b}}_{1}$, and two extreme points of ${\vec{b}}_{1}$ are on the left and right of ${\vec{b}}_{4}$, respectively. Therefore, the bisectors ${\vec{b}}_{1}$ and ${\vec{b}}_{4}$ are intersected in the constrained circle.

### 3.2. Three Steps of the Boundary Scan Algorithm

In the BS algorithm, there are three steps to construct the limited Voronoi cell. The first step is a preprocessing step to obtain bisectors within the constrained range between the generator and its neighbors in the sensing range. In the bisector set, one of the bisectors should be taken as a reference to determine other boundaries of the limited Voronoi cell. The selection of the first bisector used to initiate the algorithm deserves special attention. Since the Voronoi cell consists of the bisectors closer to its generator than to others, the bisector between the generator and its nearest neighbor would be a reasonable choice.

**Lemma** **1.** One of the boundaries of the limited Voronoi cell is on the bisector between the generator and its nearest neighbor.

**Figure 3:**
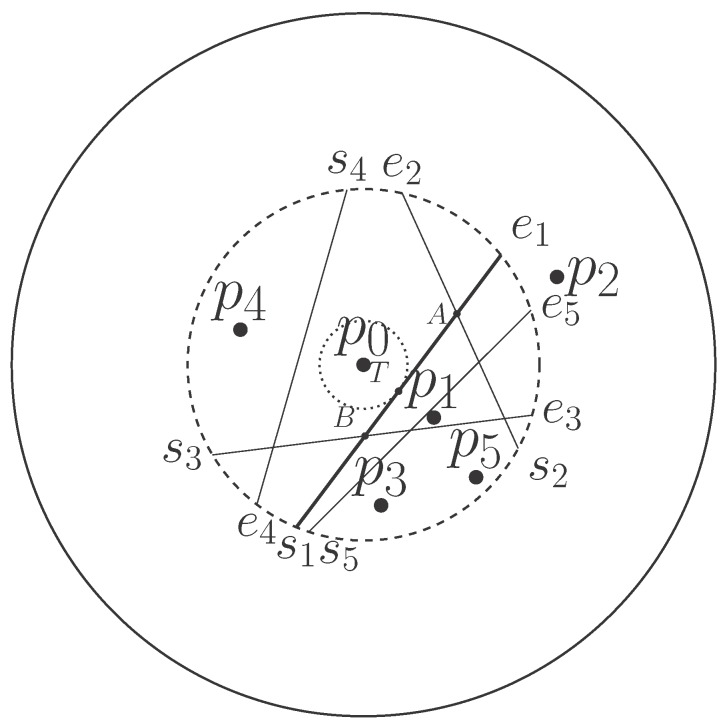
The determination of the initial bisector.

**Proof.**  A feasible bisector used to determine the validity of other bisectors should not be deleted during the entire implementation of the algorithm. Without loss of generality, given a WSN with six sensors in Figure 3, the nearest neighbor of $p_{0}$ is $p_{1}$. Their bisector ${\vec{b}}_{1}=s_{1}e_{1}$ is illustrated as a thick line segment. An auxiliary circle represented as the smallest dashed circle with the center $p_{0}$ and the radius equal to the distance from $p_{0}$ to ${\vec{b}}_{1}$ is tangent to ${\vec{b}}_{1}$ at *T*.

As the distance between $p_{0}$ and ${\vec{b}}_{1}$ is the smallest one among all the bisectors in the set $B_{0}$, there are two types of relative positions between ${\vec{b}}_{1}$ and other bisectors in the constrained circle. In the first case, other bisectors do not intersect with ${\vec{b}}_{1}$, so that they do not intersect with the auxiliary circle, such as ${\vec{b}}_{4}$ and ${\vec{b}}_{5}$. Due to the property of the Voronoi cell, the entire bisector ${\vec{b}}_{1}$ is closer to the generator than to any other bisector and should be preserved as one of the boundaries of the limited Voronoi cell. In the second case, other bisectors intersect with ${\vec{b}}_{1}$ so that intersection points are outside the auxiliary circle, such as the intersection point *A* between ${\vec{b}}_{2}$ and ${\vec{b}}_{1}$, which is on the line segment between the point of tangency *T* and extreme point $e_{1}$, as well as the intersection point *B* between ${\vec{b}}_{3}$ and ${\vec{b}}_{1}$, which is on the line segment between the point of tangency *T* and extreme point $s_{1}$. Two intersection points are always located on the different side of *T*. The part of the bisector ${\vec{b}}_{1}$ between *A* and *B* should be preserved as one of the boundaries of the limited Voronoi cell. Under no circumstance should the bisector ${\vec{b}}_{1}$ be an invalid bisector. Therefore, taking ${\vec{b}}_{1}$ as the initial bisector in the BS algorithm is an appropriate option. ☐

Then, starting from the initial bisector, other bisectors are sorted by the directional angle in a counterclockwise order. Consequently, the first step of the BS algorithm is summarized as follows.

#### 3.2.1. Step 1: The Generation of the Sorted Bisector Set

- Input: the neighbor set $P_{i}^{r}$ of $p_{i}$
- Output: the bisector set $B_{i}$ sorted by directional angles starting from that of the bisector closest to the generator
- Step 1.1: computing the intersection points between the constrained circle of $p_{i}$ and those of each sensor in its neighbor set $P_{i}^{r}$ to obtain the bisector set $B_{i}$.
- Step 1.2: computing the directional angle of each sensor in the neighbor set $P_{i}^{r}$ relative to that of the bisector closest to $p_{i}$.
- Step 1.3: sorting all the bisectors of the set $B_{i}$ according to their directional angle in a counterclockwise order.

The second step is the core of the BS algorithm. After obtaining the sorted bisector set $B_{i}$, all the bisectors will be sequentially scanned so that the boundary set of the r/2-limited Voronoi cell is determined. Since the Voronoi cell consists of points closer to the generator than to others, the part of one bisector closer to the generator is preserved as the accepted boundary of the limited Voronoi cell by comparing the relative position of two consecutive bisectors. Once the accepted boundary is farther than the currently scanned bisector, it should be removed from the boundary set of the limited Voronoi cell. Therefore, in the process of the BS algorithm, it is convenient to store the boundary set of the limited Voronoi cell in a stack.

**Figure 4:**
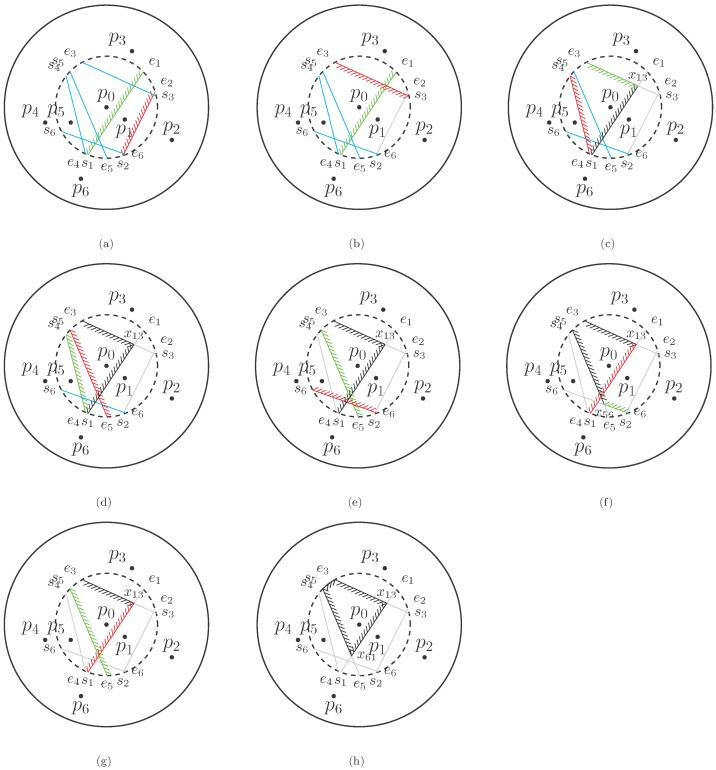
The sequential implementation of the BS algorithm.

According to the representation of bisectors in Section 3.1, the relative position between the accepted boundary stored on top of the stack and the currently scanned one along with their corresponding operation is described in detail as follows. Different types of relative positions are depicted in the corresponding subfigures of Figure 4, where all the sensors are represented as black dots. The solid and dashed circles indicate the sensing and constrained region of sensor $p_{0}$, respectively. The starting and ending points of each bisector in the set $B_{0}$ are marked along the circumference of the constrained circle. The intersection point between two bisectors is denoted as $x_{jk}$, where *j* and *k* are the indices of corresponding sensors. The green and red line segments with shade are the bisectors on top of the stack and currently scanned, respectively. The black line segments with shade are the accepted boundaries of the limited Voronoi cell stored in the stack. The blue ones without shade are the bisectors that have not been scanned yet, and the gray ones without shade are bisectors that do not constitute the r/2-limited Voronoi cell. The relative position between two bisectors and the corresponding operation to deal with this are given as follows.

1. Two bisectors are intersected:
  Two bisectors satisfy that ${\vec{b}}_{j}⋂{\vec{b}}_{k}=x_{jk}$ and $\theta_{k}-\theta_{j}<\pi$, which implies that $s_{j}$ lies on the left of ${\vec{b}}_{k}$, $e_{j}$ on the right of ${\vec{b}}_{k}$, $s_{k}$ on the right of ${\vec{b}}_{j}$ and $e_{k}$ on the left of ${\vec{b}}_{j}$ (see Figure 4b).
  In this case, the ending point of the bisector on top of the stack and the starting point of the bisector currently scanned are simultaneously replaced by the intersection point of these two bisectors, and then, the updated bisector ${\vec{b}}_{k}$ is pushed into the stack.
2. Two bisectors are intersected in a reverse order:
  Two bisectors satisfy that ${\vec{b}}_{j}⋂{\vec{b}}_{k}=x_{jk}$ and $\theta_{k}-\theta_{j}>\pi$, which implies that $s_{j}$ is on the right of ${\vec{b}}_{k}$, $e_{j}$ on the left of ${\vec{b}}_{k}$, $s_{k}$ on the left of ${\vec{b}}_{j}$ and $e_{k}$ on the right of ${\vec{b}}_{j}$. This case will occur in Figure 4 if we take ${\vec{b}}_{1}$ and ${\vec{b}}_{5}$ as two consecutive bisectors.
  No action is taken. ${\vec{b}}_{k}$ is just pushed into the stack for the subsequent operation, since, after looping back, the latter bisector will intersect with the former one as the first case.
3. Two bisectors do not intersect, and the latter is on the right of the former:
  $s_{j}$ lies on the left of ${\vec{b}}_{k}$, $e_{j}$ on the left of ${\vec{b}}_{k}$, $s_{k}$ on the right of ${\vec{b}}_{j}$ and $e_{k}$ on the right of ${\vec{b}}_{j}$ (see Figure 4a).
  Since ${\vec{b}}_{k}$ is outside the half plane generated by ${\vec{b}}_{j}$, it can not be the boundary of the Voronoi cell. Thus, the bisector on top of the stack remains unchanged, and the next candidate bisector will be scanned.
4. Two bisectors do not intersect, and the latter is on the left of the former:
  $s_{j}$ lies on the right of ${\vec{b}}_{k}$, $e_{j}$ on the right of ${\vec{b}}_{k}$ and $e_{k}$ on the left of ${\vec{b}}_{j}$ (see Figure 4d).
  Since the intersection point of two bisectors may be outside the constrained circle, once the $e_{k}$ lies on the left of ${\vec{b}}_{j}$, the scanned bisector will be closer to the generator than the one on top of the stack, i.e., the latter is outside the half plane generated by the former in the constrained circle. In this case, the bisector on top of the stack is no longer a boundary of the Voronoi cell and will be popped from the stack. Then, the second bisector in the stack becomes the one on the top and is used to compare with the currently scanned bisector.
5. Two bisectors do not intersect and lie on the left of each other:
  $s_{j}$ lies on the left of ${\vec{b}}_{k}$, $e_{j}$ on the left of ${\vec{b}}_{k}$, $s_{k}$ on the left of ${\vec{b}}_{j}$ and $e_{k}$ on the left of ${\vec{b}}_{j}$ (see Figure 4c).
  In this case, two bisectors on top of the stack and currently scanned are both valid boundaries of the limited Voronoi cell, so that ${\vec{b}}_{k}$ will be pushed into the stack.

According to the different cases and their corresponding operations described above, the second step of the BS algorithm used to generate the raw boundary set of the r/2-limited Voronoi cell is given as follows.

#### 3.2.2. Step 2: The Determination of the Boundary Set of the r/2-Limited Voronoi Cell

- Input: the sorted bisector set $B_{i}$ of $p_{i}$
- Output: the raw boundary set $VC_{i}$ of the r/2-limited Voronoi cell of $p_{i}$
- Step 2.1: initializing the stack for the boundary set $VC_{i}$ with the first element of the bisector set $B_{i}$.
- Step 2.2: sequentially scanning all the other bisectors in the set $B_{i}$ to compare with the bisector on top of the stack and obtain the boundary set $VC_{i}$ according to different cases described above.

In order to represent the r/2-limited Voronoi cell unambiguously, the boundary set obtained in the second step should be refined to a labeled vertex set. The third step of the algorithm is a post-processing step to generate the vertex set of the r/2-limited Voronoi cell by distinguishing the situation that two vertices are connected by an arc or a line segment according to the *r*-limited Delaunay neighbor set of the generator. The specific step is given as follows.

#### 3.2.3. Step 3: The Determination of the Vertex Set of the r/2-Limited Voronoi Cell

- Input: the boundary set $VC_{i}$ of the r/2-limited Voronoi cell of $p_{i}$
- Output: the labeled vertex set $V_{i}$ of the r/2-limited Voronoi cell of $p_{i}$
- Step 3.1: finding corresponding generators of the boundary set $VC_{i}$ to obtain the *r*-limited Delaunay neighbor set $DN_{i}$ of $p_{i}$.
- Step 3.2: traversing the boundary set $VC_{i}$ and labeling different types of vertices according to corresponding *r*-limited Delaunay neighbor set $DN_{i}$. Two extreme points belonging to the same bisector are labeled as line segment vertices and those belonging to two different bisectors as arc vertices. The r/2-limited Voronoi cell can be correctly represented as the labeled vertex set $V_{i}$.

In Figure 4, an instance of the whole procedure of the BS algorithm is illustrated step-by-step. Figure 4a shows the initial configuration of the bisector set. Next, sequential scanning processes are given from Figure 4b to Figure 4g, where all the boundaries of the limited Voronoi cell are gradually determined according to corresponding operations in various cases. Figure 4h is the final result of the BS algorithm.

### 3.3. Theoretical Basis of the Boundary Scan Algorithm

In the procedure of the BS algorithm applied to compute the limited Voronoi cell, there are some concepts and operations analogous to GS algorithm used to construct the convex hull of a collection of points. In the GS algorithm, basic elements to be dealt with are a point set in a plane. One of the points belonging to the convex hull is chosen as the reference to initiate the algorithm. Starting from this initial point, other points sorted by the directional angle relative to it are sequentially scanned. If the scanned point and its immediately before and after points are arranged in a convex configuration, it is added to the vertex set of the convex hull. Otherwise, it is discarded, and the next point will be scanned until the entire point set is traversed. Finally, the convex hull of a point set is precisely constructed.

Correspondingly, in the BS algorithm, basic elements for constructing the r/2-limited Voronoi cell are a collection of bisectors between the generator and its neighbors. The bisector closest to the generator is designated as the initial element to compare with other bisectors sorted by the directional angle relative to it. The property that the limited Voronoi cell consists of points closer to its generator than to others is used to determine the validity of the scanned bisector. After traversing the bisector set, the limited Voronoi cell is effectively generated. In other words, the correctness and effectiveness of the BS algorithm come from the GS algorithm.

Due to the distributed nature of WSNs, the feasibility of the limited Voronoi partition of a plane is ensured by the sufficiency of the information obtained by each sensor and the correctness of the algorithm used to autonomously compute each limited Voronoi cell. First of all, because the *r*-limited Delaunay graph is spatially distributed over the *r*-disk graph, the latter encodes enough information to generate the r/2-limited Voronoi partition of a WSN. Therefore, for each sensor, the position information of other sensors in its sensing range *r* is sufficient for each sensor to compute its r/2-limited Voronoi cell independently. Then, Lemma 1 guarantees that it is appropriate to choose the bisector between the generator and its nearest neighbor as the first bisector to initiate the BS algorithm. What is more, all the operations in the BS algorithm strictly comply with the property that the Voronoi cell consists of the points closer to the generator than to the others. As a result, at each step of the scanning process, the bisector on top of the stack and the one currently scanned are properly handled in the corresponding cases described in Section 3.2. Finally, the extreme points of boundaries of the r/2-limited Voronoi cell are labeled as the vertices belonging to an arc or a line segment unambiguously with the help of the *r*-limited Delaunay neighbor set.

### 3.4. Computational Complexity of Boundary Scan Algorithm

Since the *r*-limited Delaunay graph is spatially distributed over the *r*-disk graph, the computational complexity of the BS algorithm is closely related to the number of neighbors in the *r*-disk graph. Assuming a sensor has *m* neighbors in its sensing range, the analysis of the computational complexity of the BS algorithm with the aforementioned three steps is given in Table 1. In the first two sub-steps of the first step and second step, the whole neighbor set with *m* elements is traversed. In the third step, the refined vertex set has the number of operations less than or equal to *m* in that some invalid bisectors have been removed from the bisector set after the first two steps. In the third sub-step of the first step, sorting *m* bisectors has the highest computational complexity. As a result, the computational complexity of the BS algorithm as a whole is O(mlogm). Due to the equivalence of the BS and GS algorithm, the BS algorithm reaches the lower bound on the computational complexity of the algorithms used to solve this problem.

Meanwhile, the RCVC algorithm proposed in [12] is applied to solve a similar problem, where the *r*-limited Voronoi cell of a robot is computed based on the position information of the robots in the 2r communication range. Its computational complexity is analyzed in Table 2. There are also three steps in the RCVC algorithm. Except for constructing and sorting the set of polar angles $\Theta_{i}$, which consist of polar angles of all the extreme points of bisectors in $B_{i}$, the first step of the RCVC algorithm is the same as the corresponding one in the BS algorithm. In the second step, invalid bisectors are removed from the bisector set $B_{i}$ by constructing the set of polar angles $\Theta_{i}^{diff}$ consisting of polar angles of the extreme points of other bisectors lying between the bisector ${\vec{b}}_{i}$. Notably, the computational complexity of this step is not correctly analyzed in that paper. For every bisector ${\vec{b}}_{i}$, it will traverse all the other bisectors to check their validity in the worst case, so that the computational complexity of this step is actually $O(m^{2})$ rather than O(m), which is claimed in that paper without considering the computational complexity of the sorting operation. Therefore, the entire RCVC algorithm has a higher computational complexity than the BS algorithm.

For comparison, the centralized algorithms used to construct the exact Voronoi diagram of a set of *n* points, such as the divide-and-conquer algorithm [20] and the sweep line algorithm [5], have a computational complexity equal to O(nlogn). Thus, the algorithm applied in the ASR algorithm, i.e., the EVT algorithm, has a computational complexity O(mlogm) to compute the limited Voronoi cell of a sensor with *m* neighbors. Although the EVT algorithm is not the most efficient method to compute the r/2-limited Voronoi cell, it provides a lower bound of the computational complexity of the algorithm used to compute the limited Voronoi cell and can be regarded as a benchmark to verify the correctness of the proposed algorithm.

## 4. Simulation Studies

### 4.1. Simulations of Boundary Scan Algorithm Compared with Existing Algorithms

Although the algorithm of the limited Voronoi partition should be implemented on a WSN platform, it is easy and sufficient to demonstrate the advantages of the proposed algorithm over existing ones by performing simulations on a general PC platform. The correctness and effectiveness of the BS algorithm are verified by extensive numerical simulations, in which algorithms are coded by Python 2.7.12 and implemented on Windows 10 with Intel Core i7-4770 at 3.4 GHz and 8 GB memory. It is expected that the lower computational complexity of the proposed algorithm and the simple mechanism of information exchange among nodes by means of the passive sensing or one-hop communication will lead to a lower energy consumption and small number of sending and receiving messages. The advantages of our proposed algorithm are platform-independent.

**Figure 5:**
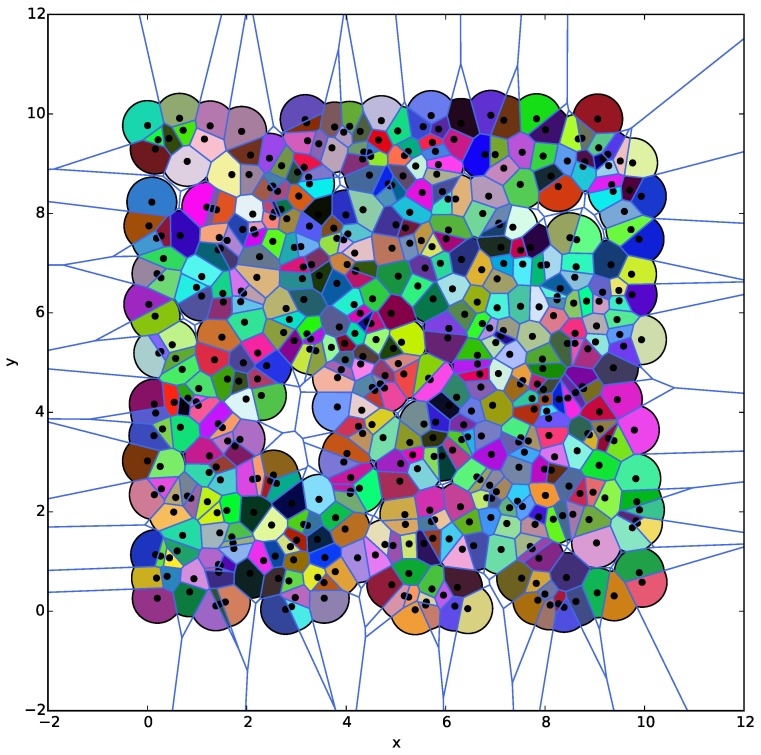
The comparison of the exact and r/2-limited Voronoi partition of the same WSN.

First, we compare the limited Voronoi partition obtained by the BS algorithm implemented on all the sensors with the exact Voronoi tessellation of a WSN generated by a centralized algorithm. For a WSN consisting of 500 sensors with a fixed sensing range r=1 and uniformly distributed on a 10×10 plane, the exact and the r/2-limited Voronoi partition of this network computed by aforementioned two algorithms are depicted together in Figure 5, where the exact Voronoi cells are polygons composed of a set of line segments as their boundaries, while the corresponding r/2-limited Voronoi cells are the regions filled with different colors. The white parts near the boundary and in the sparse area of the plane are the regions that cannot be covered by the r/2-limited Voronoi cells. It is obvious that the results generated by two algorithms are similar except for several Voronoi cells since the region partitioned by the WSN with a limited sensing range is not the entire plane, but the union of all the constrained circles with a half sensing radius. Therefore, the correctness of the BS algorithm is easily verified.

Next, the performance of the proposed BS algorithm and that of the existing RCVC algorithm are compared through the computational time spent by two algorithms to compute the r/2-limited Voronoi partition of the same WSN with various configurations. According to the analysis in Section 3.4, the number of neighbors of a sensor is closely related to the computational complexity of both the BS and RCVC algorithm. Moreover, a WSN is usually a dynamical system in practical applications. Sensors should accomplish some tasks by means of cooperative motions so that the configuration of the network will change dynamically. In different scenarios, the density of the WSN is variable, so is the average number of neighbors of each sensor. Therefore, the time efficiency of two algorithms is compared through computing the limited Voronoi partitions of a WSN with the increasing density of sensors.

For a WSN consisting of 500 sensors with a fixed sensing range r=1, the length of the region, where all the sensor are deployed, is varied from one to 10 with a unit interval. The relationship between the density of the WSN ρ and the length of the region *L* is $\rho =N/L^{2}$. For each density, the total computational times spent by all the sensors to compute their Voronoi cells through implementing the BS and RCVC algorithm are averaged for 10 randomly-generated configurations, respectively.

**Figure 6:**
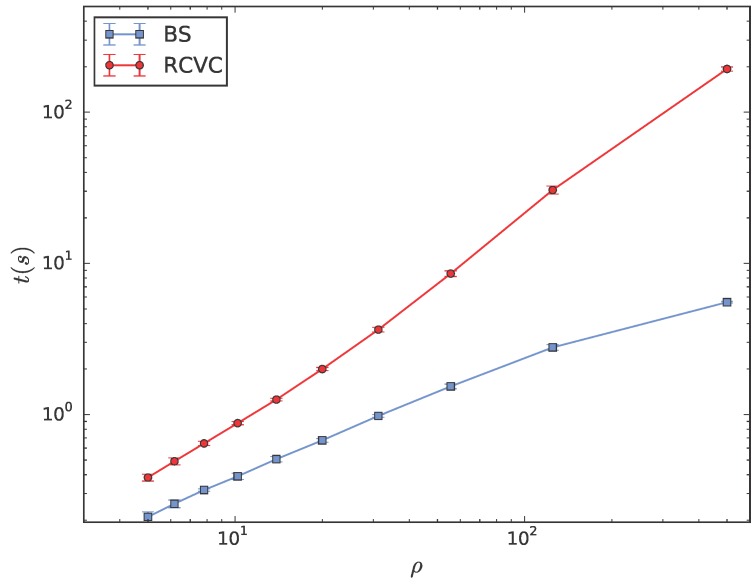
The computational time of the BS and RCVC algorithm versus the increasing density of WSNs.

Two curves of the computational time of the BS and RCVC algorithm with the increasing density of WSNs are illustrated in Figure 6, where the horizontal and vertical axes represent the density and the computational time, respectively. For a better illustration, curves are plotted in the dual-logarithmic coordinates. The blue and red curves of the computational time are generated by the BS and RCVC algorithm, respectively. It takes a longer time to compute the limited Voronoi cell with the increasing density of WSNs since more sensors are included in the neighbor set of the *r*-disk graph. In general, due to the classic ASR algorithm, the problem of computing the limited Voronoi cell with a large-scale neighbor set can be decomposed into multiple subproblems with a small number of neighbors. Moreover, the most common configuration of the WSN has a moderate density, i.e., each sensor has tens of neighbors in its sensing range. In this situation, the computational time of the RCVC algorithm is three- to six-times longer than that of the BS algorithm. In the low-density situation, each sensor has a relatively small number of neighbors in its sensing range. The BS algorithm runs just one time faster than the RCVC algorithm. Although it is less likely to deal with an extremely high-density WSN in applications, we should not ignore this worst case, where the difference between the time efficiency of two algorithms increases up to 70 times. In all the scenarios, the BS algorithm provides a full-range improvement in computational time.

Then, in order to demonstrate the improvement of the ASR algorithm after introducing the BS algorithm, the computational times of the BS algorithm implemented individually and embedded into the framework of ASR algorithm are compared with corresponding existing algorithms in the following simulations. For one thing, since the BS algorithm is used to replace the EVT algorithm used in the standard ASR algorithm, the computational time of the BS and EVT are compared first. For another, both the BS and EVT algorithms are applied in one of the key steps in the ASR algorithm. Hence, two algorithms combined with the ASR algorithm (ASR+EVT and ASR+BS) are implemented under the same condition. Since the BS and EVT algorithms are usually used to construct the limited Voronoi cell of a sensor with a small number of neighbors, we generated a typical configuration of a WSN consisting of 30 sensors uniformly distributed in a 3×3 plane, where the average number of neighbors of each sensor is about nine.

**Figure 7:**
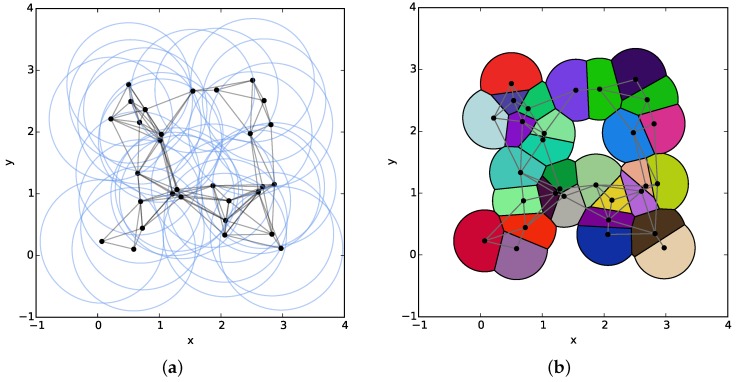
The *r*-disk graph and corresponding r/2-limited Voronoi diagram of a WSN. (**a**) The *r*-disk graph. (**b**) The r/2-limited Voronoi diagram.

An example of such WSNs is illustrated in Figure 7, where the *r*-disk graph and the corresponding r/2-limited Voronoi diagram are depicted in two subfigures, respectively. We randomly generate 100 such configurations to compare the computational time spent by two individual and two combined algorithms. The average times with standard deviations are given in Table 3. The result indicates that the BS algorithm is superior to its counterpart in both cases. In addition, when observing the second and fourth row of this table, we notice an interesting result that the BS algorithm performs even better than the ASR+BS algorithm to compute the limited Voronoi partition of such a configuration of the WSN.

**Figure 8:**
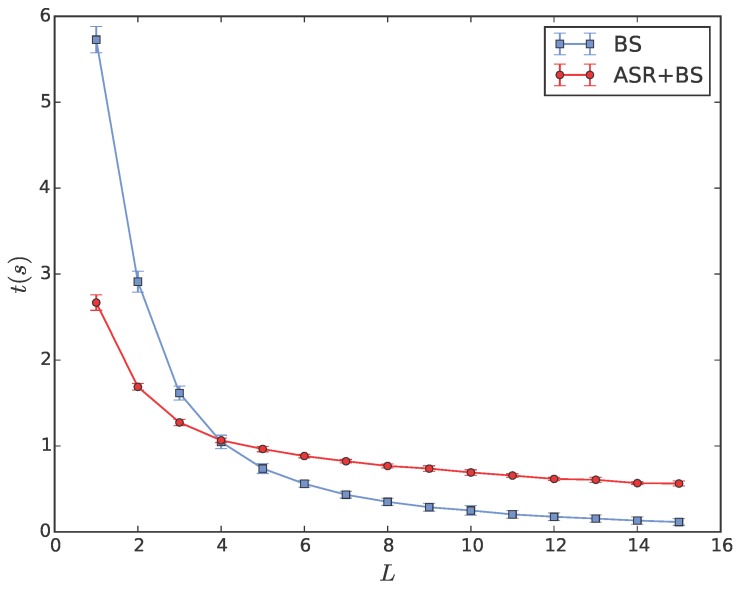
The computational time of the BS and ASR+BS algorithm versus the increasing side length of the region.

Finally, motivated by the result of the previous simulation, we carry out a detailed simulation to compare the computational time of the BS algorithm implemented individually with that embedded into the ASR algorithm in various scenarios. For a WSN consisting of 500 sensors with a fixed sensing range r=1, all the sensors are uniformly distributed in a square region whose side length is varied from 1 to 15 with a unit interval so that WSNs with different densities are generated correspondingly. For each side length of the region, we randomly generate 100 networks to compare the computational time of two algorithms. The means and standard deviations of the computational times spent by two algorithms with the increasing length of regions are depicted in Figure 8, where the computational times of two algorithms are almost equal at L=4. Obviously, this critical side length of the region corresponding to the critical density of WSNs can be used to determine the applicability of two algorithms qualitatively. The ASR+BS algorithm is more efficient for a network with a relatively high density, where most limited Voronoi cells are the same as the exact ones without arc boundaries. Consequently, the termination condition of the ASR algorithm can be quickly achieved without traversing the large-scale neighbor set as the BS algorithm does. On the contrary, the BS algorithm will perform better for a network with a relatively low density, where the number of sensors in the sensing range is small and the limited Voronoi cell is usually enclosed by arc and line segment boundaries. The ASR algorithm will not terminate until the adjustable sensing range is enlarged to the actual one. The computational time spent in the last step of the ASR algorithm is equal to that spent in the entire BS algorithm. Therefore, the total time of the ASR algorithm is no doubt longer than that of the BS algorithm. In practical applications, it is more reasonable to consider a tradeoff between the iterated, multi-step ASR algorithm and the traversed, single-step BS algorithm. For instance, each sensor should first evaluate the local density in its sensing range and then decides which algorithm is suitable for the current situation. This problem is worthy of further investigation.

In practical applications, the distributed algorithm of the limited Voronoi partition of a plane is generally applied to the dynamic simulation of multi-robot systems [21], sensor networks [10] and self-propelled particles [22]. In these systems, each individual will first compute its own limited Voronoi cell as the dominant region autonomously and then plan its motion for the next step according to the obtained limited Voronoi cell. In particular, the entire dynamic simulation of collective motion of self-propelled particles will incur a heavy computational burden. For one thing, the scale of the particle group is quite large, up to $10^{4}$ in general. During the evolution, the formation of the particle group may change drastically. Thus, the geometric configurations with which the ASR algorithm cannot efficiently deal will frequently occur. For another, it will take a long time to implement the simulation of collective motion of self-propelled particles, $10^{5}$ steps in general, in order to avoid memory effects of the initial condition. The total time of the entire simulation would be reduced substantially, even though there was only a small improvement in the computation time of the limited Voronoi cell in each step. Therefore, the improvement in the time efficiency provided by the proposed BS algorithm is significant in practical applications.

### 4.2. Simulations of Boundary Scan Algorithm Combined with the Localization Algorithm

**Figure 9:**
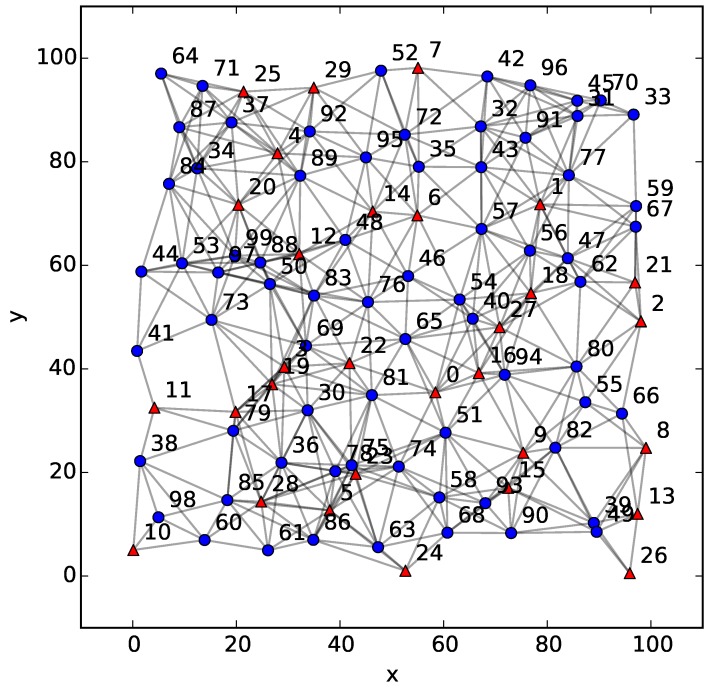
The configuration of the WSN used with the DV-Hop algorithm.

For WSNs used in practice, the position of each node is generally not known in advance, but should be estimated by means of some localization algorithm. In order to demonstrate the utility of the proposed BS algorithm in the field of WSNs, a simulation combining the BS algorithm with the DV-Hop algorithm, a widely-used localization technique for ad hoc and wireless sensor networks [18], is performed to generate the limited Voronoi partition of a WSN based on the estimated positions of nodes. For a WSN consisting of 100 sensors with the same sensing and communication range r=20 and uniformly distributed on a 100×100 plane, the configuration and the connectivity relationship of this network are shown in Figure 9, where 30% of the nodes are the anchor nodes with known positions and represented by red triangles, and other normal nodes to be located are blue circles. Two nodes that can exchange information with each other are connected by a gray line as the communication link.

**Figure 10:**
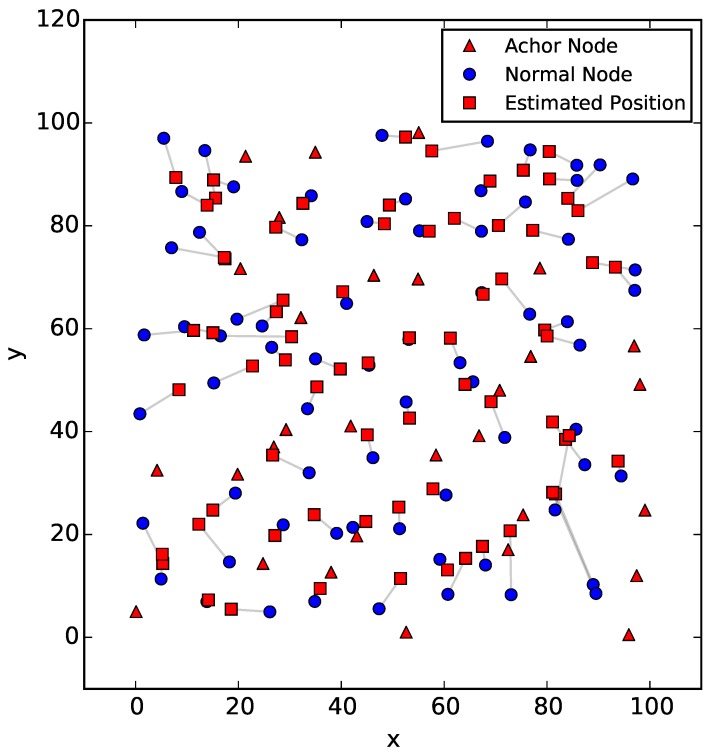
The true and estimated positions of nodes in the WSN.

The simulation result generated by the DV-Hop algorithm is given in Figure 10, where the anchor and normal nodes are still represented by red triangles and blue circles, respectively. The estimated positions of the normal nodes are marked as red squares. The gray lines indicate the deviations between the true and estimated positions of nodes.

**Figure 11:**
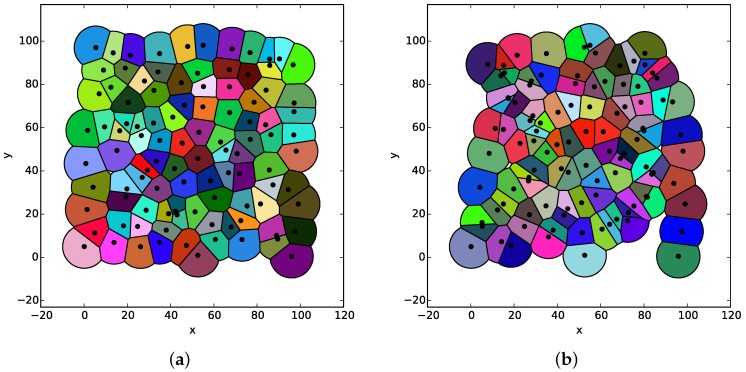
The comparison with the limited Voronoi partition of the WSN with exact and estimated node positions. (**a**) The limited Voronoi partition of the WSN based on exact node positions. (**b**) The limited Voronoi partition of the WSN based on estimated node positions.

Based on the true and estimated positions of nodes, two limited Voronoi partitions of the WSN generated by the BS algorithm are depicted in Figure 11. It is obvious that the accurate localization of each node plays an important role in the computation of the limited Voronoi partition.

**Figure 12:**
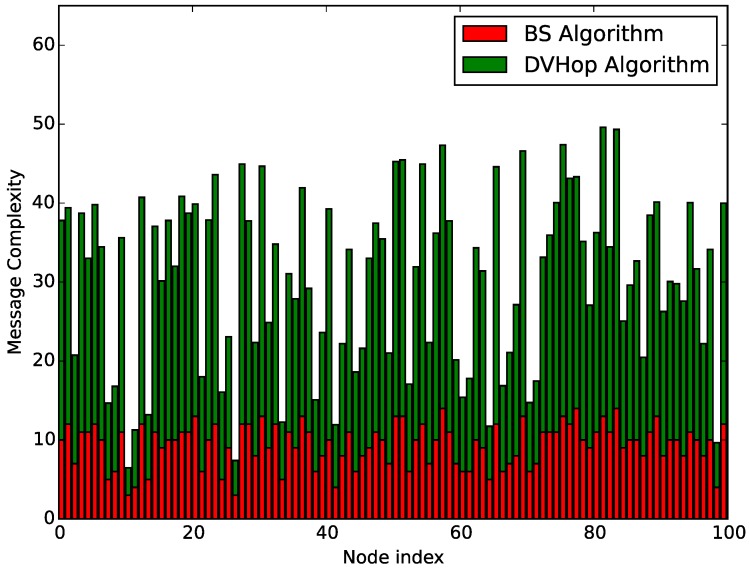
The number of transmitted messages of each node in the BS combined with the DV-Hop algorithm.

In order to evaluate the cost of transmitted messages of the proposed algorithm, the number of sending and receiving messages of each node in the distributed implementation of the BS combined with the DV-Hop algorithm for the WSN given above is regarded as a measurement of the message complexity. The result shown in Figure 12 indicates that the anchor nodes with known positions have a relatively low message complexity, while the normal nodes having more neighbors have to exchange necessary information with their neighbors more times to achieve localizations and computations. Moreover, anchor nodes will broadcast their information twice in the DV-Hop algorithm, while all the nodes implementing the BS algorithm just need to transmit their positions to their neighbors once. The communication cost in the BS algorithm accounts for a small proportion of the total cost. In addition, the energy consumption of the network used to generate the limited Voronoi partition is related primarily to the number of communications among nodes. By ignoring some minor factors such as the computational cost and idle power of each node, we believe that the number of information exchanges among nodes could indicate the energy consumption for computing the limited Voronoi partitions of the WSN to some extent [23]. When the hardware features of nodes such as the CPU type and transmit/receive power are given, referring to the package size and transfer rate defined in the IEEE 802.15.4 protocol [24], the energy consumption of each node can be exactly calculated.

## 5. Conclusions

For a wireless sensor network, the boundary scan algorithm is proposed in this paper to compute the limited Voronoi cell of each sensor with a limited sensing range in order to generate the limited Voronoi partition of a plane in a distributed manner. Inspired by GS algorithm used to construct the convex hull of a collection of points, through sequentially scanning bisectors between a sensor and its neighbors, comparing the relative position of two consecutive bisectors and implementing corresponding operations, each sensor can construct its limited Voronoi cell autonomously. The computational complexity of the BS algorithm is lower than that of the RCVC algorithm and reaches the lower bound of the computational complexity of equivalent algorithms. Furthermore, the proposed algorithm is an improvement of the key step in the ASR algorithm and is also more efficient than the ASR algorithm for low-density WSNs. The correctness and time efficiency of the proposed BS algorithm are demonstrated by extensive numerical simulations.

Since our paper is just involved with a theoretical algorithm extracted from the practical applications of WSNs, the simulation studies are implemented merely on a general PC platform rather than an emulation environment or a real WSN at present. The implementation of the proposed algorithm on a practical WSN platform is one of the indispensable works in the future.

## Figures and Tables

**Table 1:** The computational complexity of the Boundary Scan (BS) algorithm.

Step	Operation	Complexity
1	Computing bisector set $B_{i}$	O(m)
	Finding an initial bisector	O(m)
	Sorting bisector set $B_{i}$ by directional angles	O(mlogm)
2	Scanning sorted bisector set $B_{i}$	O(m)
3	Computing vertex set $V_{i}$	$O\left(m^{\prime }\right),m^{\prime }\leq m$

**Table 2:** The computational complexity of the Range-Constrained Voronoi Cell (RCVC) algorithm.

Step	Operation	Complexity
1	Computing bisector set $B_{i}$ and polar angle set $\Theta_{i}$	O(m)
	Finding an initial bisector	O(m)
	Sorting bisector set $B_{i}$ and polar angle set $\Theta_{i}$	O(mlogm)
2	Removing redundant bisectors according to polar angle set $\Theta_{i}^{diff}$	$O(m^{2})$
3	Computing vertex set $V_{i}$	$O\left(m^{\prime }\right),m^{\prime }\leq m$

**Table 3:** The computational time of the individual Exact Voronoi Tessellation (EVT) and BS algorithms and those embedded in the framework of the Adjust-Sensing-Radius (ASR) algorithm

Algorithms	Computational Times (s)
EVT	0.02703±0.00926
BS	0.00574±0.00096
ASR+EVT	0.06043±0.00537
ASR+BS	0.01177±0.00190
